# Diosmetin inhibits cell proliferation and promotes apoptosis through STAT3/c-Myc signaling pathway in human osteosarcoma cells

**DOI:** 10.1186/s40659-021-00363-1

**Published:** 2021-12-18

**Authors:** Rende Ning, Guang Chen, Run Fang, Yanhui Zhang, Wenjuan Zhao, Feng Qian

**Affiliations:** 1 Department of Orthopaedics, The Third Affiliated Hospital of Anhui Medical University, 390 Huaihe Road, Hefei, 230031 China; 2 Department of Otolaryngology Head and Neck Surgery, Shanghai General Hospital, 85 Wu Jin Road, Shanghai, 200080 China; 3 Engineering Research Center of Cell & Therapeutic Antibody, Ministry of Education, School of Pharmacy, Shanghai Jiao Tong University, 800 Dongchuan Road, Shanghai, 200240 China

**Keywords:** Diosmetin, Osteosarcoma, Signal transducer and activator of transcription 3 (STAT3), Cell proliferation, Apoptosis

## Abstract

**Background:**

Diosmetin is a bioflavonoid compound naturally abundant in citrus fruits. It is found to perform a variety of activities, while its antitumor property in osteosarcoma, a malignant tumor with unmet clinical treatment, remained unknown.

**Methods:**

Colony formation assay, cell cycle analysis and apoptosis analysis were conducted respectively to observe the effect of diosmetin on cell proliferation and apoptosis in human osteosarcoma cells. Western blot and immunoprecipitation were used to detect the expression of apoptotic molecules and activation of STAT3/c-Myc pathway in Saos-2 and U2SO cells.

**Results:**

Diosmetin significantly inhibited cell proliferation, induced cell cycle arrest at G2/M phase and promoted cell apoptosis in both Saos-2 and U2SO cells. Moreover, Diosmetin downregulated the expression of anti-apoptotic protein Bcl-xL while upregulated the levels of pro-apoptotic proteins including cleaved Caspase-3, cleaved-PARP and Bax. Furthermore, diosmetin dose-dependently inhibited STAT3 phosphorylation, reduced the expression of its downstream protein c-Myc and impeded the interaction between STAT3 molecules.

**Conclusions:**

These results suggest that diosmetin exerts anti-osteosarcoma effects by suppressing cell proliferation and inducing apoptosis via inhibiting the activation of STAT3/c-Myc signaling pathway, which provide the possibility for diosmetin to be a chemotherapeutic candidate for osteosarcoma.

## Background

Osteosarcoma is one of the most common malignant bone tumors among children and adolescents and mostly occurs in the metaphyses of limb long bones. Despite significant advances in surgery and multiagent chemotherapy in recent decades, the 5-year survival rate of osteosarcoma is still low [1, 2]. In addition, present chemotherapy drugs for osteosarcoma have high cytotoxicity and large side effects [1, 2]. Almost half of the osteosarcoma survivors have to suffer at least one adverse consequence from antitumor therapy. Therefore, finding new effective drugs with low toxicity to treat osteosarcoma is of great clinical significance.

Signal transducer and activator of transcription 3 (STAT3) is an important transcription factor with multiple biologic activities in cell proliferation, survival, apoptosis, and differentiation in many cell types [3]. Activated STAT3 usually forms a dimer, then rapidly translocate from cytoplasm into the nucleus, triggering the transcription of the downstream genes involved in tumor proliferation and survival [4]. It has been demonstrated STAT3 plays a vital role in osteosarcoma development and thus might be a promising target for drug discovery of human osteosarcoma [5–7]. Over the past 20 years, several therapeutic agents targeted STAT3 signaling have been developed including small molecules, peptides and peptoids [8]. However, most STAT3 inhibitors are usually accompanied by severe adverse events like high cytotoxicity and poor pharmacokinetic properties, which renders the drug-making properties unsatisfied. In contrast, agents from natural sources are safety, efficacy and immediate availability, thus are considered the best sources of drugs and drug leads for novel drug discovery. A variety of natural products with STAT3 inhibitory activity such as curcumin, resveratrol and JSI-124 (cucurbitacin I), have been isolated from natural products, and most are branched and flavonoids [9–12].

Diosmetin, is a bioflavonoid compound extracted from the herbaceous plant bedstraw and citrus with broad activities, including antioxidation, anti-inflammation, anti-apoptosis, anti-virulence and antinociception [13–16]. Notably, diosmetin did not show any detectable adverse effects in an acute toxicity study, suggesting that it may be safe for use in the human body [16]. In recent years, several studies found that diosmetin exerted antitumor effect on liver cancer and breast cancer through inhibiting tumor proliferation, inducing tumor cell apoptosis and regulating tumor cell cycles [17, 18]. While its antitumor role in osteosarcoma cells remained unknown. Moreover, the antitumor mechanisms of diosmetin varied in different tumors, and none of which have been clearly elucidated. Herein, we evaluated the anti-osteosarcoma effects of diosmetin on proliferation, cell cycle progression and apoptosis in human osteosarcoma cell lines Saos-2 and U2SO. Furthermore, we revealed that the inhibition of STAT3 signaling might be involved in diosmetin-induced apoptosis, G2/M cell cycle arrest as well as the inhibition of cell proliferation.

## Materials and methods

### Reagents

Diosmetin (purity > 98%) and WP1066 (STAT3 inhibitor) were purchased from Sigma-Aldrich Co (St. Louis, MO, USA). Dulbecco's modified eagle medium (DMEM), trypsin, phosphate buffered saline (PBS), penicillin and streptomycin were all purchased from Thermo Fisher Scientific (Grand Island, NY, USA). Fetal bovine serum was obtained from Sijiqing Biological Engineering Materials Co. Ltd (Hangzhou, China). Interleukin-6 (IL-6) was acquired from Peprotech (Rocky Hill, NJ, USA). The primary antibodies against the cleaved-PARP, cleaved-caspase-3, Bcl-xL, Bax, STAT3, p-STAT3, c-Myc, β-actin, hemagglutinin (HA) and Flag tags were supplied from Cell Signaling Technology (USA). IP kit, Wright-Giemsa staining kit, Annexin V-FITC cell apoptosis detection kit and cell cycle and apoptosis analysis kit were purchased from Beyotime Institute of Biotechnology (Shanghai, China).

### Pharmaceutical preparation

Diosmetin powder was dissolved in dimethyl sulfoxide (DMSO) to prepare a stock solution of 20 mM and stored in aliquots at − 80 °C for long-term. The solution was diluted to different working concentrations with DMEM for experiments as needed.

### Cell culture

Human osteosarcoma cancer cell lines Saos-2, U2SO were obtained from American Type Culture Collection (ATCC). Saos-2 and U2SO cells were cultivated in DMEM medium containing 10% fetal bovine serum and 1% penicillin–streptomycin and routinely cultured in a humidified incubator with 5% CO_2_ at 37 °C.

### Colony formation assay

Made single cell suspension when osteosarcoma Saos-2 and U2SO cells reached 70% confluence, that is, roughly 70% of the growth surface was covered by cells seen under the microscope. Counted and adjusted the cell concentration to 1 × 10^3^/mL and then seeded in 6-well plates (500 cells/well), evenly disperse the cells. The cells were cultivated in normal DMEM medium at 37 °C for 6 h before diosmetin treatment. Thereafter, cells were incubated with different concentrations of diosmetin (0, 0.1, 0.3 and 0.9 μM) in DMEM containing 10% FBS and incubated for 10 days. 0 μM diosmetin, i.e., DMSO (final concentration 0.045%) was used as a control. Normally, the colonies were visible to the naked eye at day 7 and reached optimal clones of about 200 cells in each clone at day 10. The culture medium was discarded at day 10 after diosmetin treatment and the colonies were softly washed twice with PBS. Fixed the colonies with methanol at room temperature for 15 min. Then, discard the fixative and stained the colonies with Wright-Giemsa staining kit for 20 min, rinsed the staining solution and let them dry in the air for 1 h. The numbers of colonies in different wells were counted and then performed statistical analysis to analyze the effect of diosmetin on cell proliferation.

### MTT assay

For the preparation of stock solution (5 mg/mL), 50 mg of 3-(4,5-dimethylthiazol-2-yl)-2,5-diphenyltetrazolium bromide (MTT) powder was added to 10 mL of sterile PBS, mixed by vortexing until dissolved. Saos-2 or U2SO cells at 70% confluence were collected separately and adjusted to 1 × 10^5^ /mL, seeded (1 × 10^4^ cells/well) in a 96-well plate overnight. Next day, the culture media were changed the cells were treated with different concentrations of diosmetin (0, 10, 30 and 90 μM) for 24 h. 0 μM diosmetin, i.e., DMSO (final concentration 0.045%) was used as a control. 20 μL of MTT solution were added to each well and incubated at 37 °C for another 4 h. Absorbance was measured at 570 nm by a microplate reader.

### Cell cycle analysis

Saos-2 or U2OS cells at 70% confluence were seeded in 6—well plates at a density of 4 × 10^5^ cell per well, cultured overnight, synchronized in serum-free medium for 24 h, and then treated with different concentrations diosmetin (0, 10, 30 and 90 μM) in 0.045% DMSO for 24 h. The cells were harvested, gently rinsed and resuspended with ice-cold PBS to obtain a mono-dispersed cell suspension, with minimal cell aggregation. And then, the cells were fixed with 70% ethanol at 4 °C for 24 h. After centrifugation at 4 °C at 1000 r/min for 4 min, the precipitated cells were washed twice, with pre-cooled PBS, then treated with 10 μg/ml RNase with 0.1% Triton X-100 at room temperature for 30 min and stained with 50 μg/ml propidium iodide (PI) staining solution in dark for 5 min. Red PI fluorescence was detected by a flow cytometry (LSRFortessa TM X-20; BD Biosciences, San Jose, NJ, USA). The DNA contents in cells were measured by the flow cytometer and the proportions of each phase (G0/G1, S, and G2/M) in the cell cycle were analyzed with its equipped cell cycle analysis software.

### Apoptosis analysis

Saos-2 or U2OS cells at 70% confluence were seeded in 6-well plates at a density of 4 × 10^5^ cell per well and cultured overnight before incubation with different concentrations diosmetin (0, 10, 30 and 90 μM) dissolved in DMSO (final concentration 0.045%) for 24 h. Then harvested and washed the cells twice with ice-cold PBS. After resuspending the cells in 195 μL Annexin V-FITC binding solution, added 5 μL Annexin V-FITC and all 10 μL PI staining solution, mixed gently and incubated for 10 min in dark at room temperature. Cell apoptosis was immediately detected and analyzed by a flow cytometry (LSRFortessa TM X-20; BD Biosciences, San Jose, NJ, USA). Annexin V-FITC was green fluorescence and PI was red fluorescence. PI-negative and annexin-positive cells were early apoptotic cells (lower right quadrant), both PI-and annexin-positive cells were late apoptotic cells (upper right quadrant) while PI-and annexin-double negative cells were normal cells (lower left quadrant).

### Western blot

Saos-2 or U2SO cells (1 × 10^6^/well) were seeded in 6-well plates and culture overnight before incubation with different concentrations diosmetin (0, 10, 30 and 90 μM) for 24 h. The cells were gently washed with pre-cooled PBS, fully lysed in 1 × loading buffer to extract protein. and heated for 10 min at 99 °C. Protein lysates were heated at 99 °C for 10 min before fractionated by electrophoresis in 10% or 12% sodium dodecyl sulfate polyacrylamide gel electrophoresis (SDS–PAGE) and transferred to nitrocellulose membranes. The membranes were then blocked with 5% non-fat milk at room temperature for 2 h, incubated with the corresponding primary antibodies at 4 °C overnight, washed three times with TBST. Then incubated horseradish peroxidase (HRP)-linked secondary antibody at room temperature for 2 h followed by another three-time washing. The proteins were visualized with enhanced chemiluminescence (ECL) reagents and exposed to X-ray film. Image-J software was used to analyze the gray value of the protein band. The ratio of the gray value of the target protein band to the gray value of the β-actin band was the relative expression of the target protein.

### Immunoprecipitation

Saos-2 cells (1 × 10^6^/well) were seeded in 6-well plates and culture overnight before incubation with different concentrations diosmetin (0, 10, 30 and 90 μM) for 24 h. The cells were washed with pre-cooled PBS and lysed on ice for 30 min with cell IP lysis buffer (containing protease inhibitor). After centrifugation at 1000 r/min at 4 °C for 4 min, the deposited cell lysates were incubated with corresponding antibody and protein A/G-beads at 4 °C overnight in a slowly shaking shaker. The beads were then eluted with PBS and protein was released by adding loading buffer. Immunoprecipitated samples and total cell lysates fractions (input) were analyzed by immunoblotting.

### Statistical analysis

In this study, the experimental data were the results of at least three independent repeated experiments, and were expressed as mean ± SD. one-way analysis of variance ANOVA and Tukey test was used to evaluate the difference group between the indicated values (GraphPad Prim 8 software) and * P < 0.05, ** P < 0.01 and *** P < 0.001 indicated statistical significant difference.

## Results

### Diosmetin inhibited the proliferation of osteosarcoma cells

**Fig. 1:**
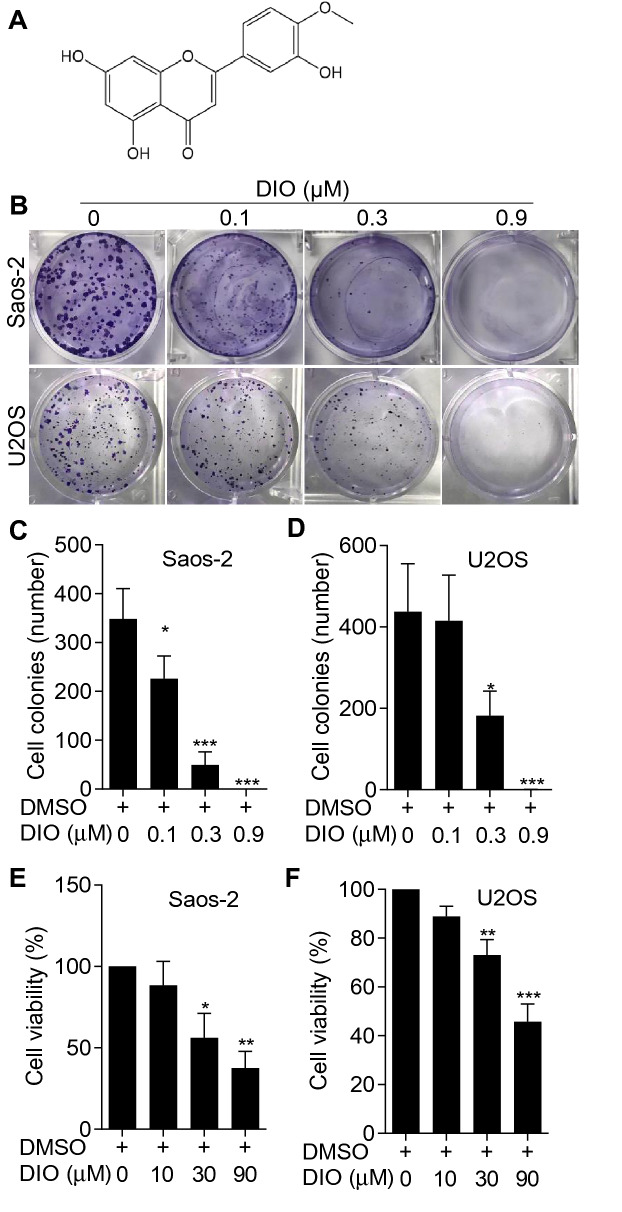
Diosmetin inhibited the proliferation and cell viability of osteosarcoma Saos-2 and U2SO cells. **A** Chemical structure of diosmetin. **B** Representative images of cell colonies of Saos-2 and U2SO cells from six-well plates in which cells were treated with 0, 0.1, 0.3 and 0.9 μM of diosmetin (DIO) for 24 h by colony formation assay. **C**, **D** Quantification of colony number in diosmetin-treated Saos-2 (**B**) or U2SO (**C**) cells. **E**, **F** The cell survival rate of Saos-2 (**D**) and U2SO (**E**) cells which were treated with 0, 10, 30 and 90 μM of diosmetin respectively in 96-well plate were quantitatively analyzed by MTT assay. The results were shown as the mean ± SD of three independent experiments (n = 3, *P < 0.05, **P < 0.01 and ***P < 0.001 vs. DMSO control)

In order to identify the anti-tumor activity of diosmetin, 3′,5,7-trihydroxy-4′-methoxyflavone (Fig. 1A), in osteosarcoma cells, we investigated its anti-proliferative role using colony formation assay on human osteosarcoma Saos-2 and U2SO cell lines. As shown in the Fig. 1B, the proliferation of both Saos-2 and U2SO cells was gradually inhibited by diosmetin with increasing concentration. Quantification analysis of the colony numbers of both Saos-2 and U2SO cells revealed that diosmetin hindered cell proliferation of osteosarcoma cells in a dose-dependent manner (Fig. 1C and D) and diosmetin at 0.9 μM for 10 days almost completely inhibited cell proliferation of both Saos-2 and U2SO cells (Fig. 1B, C, and D), representing a strong inhibitory effect of diosmetin on osteosarcoma cells. Meanwhile, the cellular toxicity of diosmetin on human osteosarcoma Saos-2 and U2SO cells were detected by MTT assay after the cells were treated with different concentrations of diosmetin for 24 h. Figure 1E and F showed that diosmetin dose-dependently inhibited the cell viability of both Saos-2 and U2SO cells, and had significant cellular toxicity at 30 and 90 µM. Taken together, diosmetin exerted obvious inhibitory effects on the proliferation and cell viability of osteosarcoma cells.

### Diosmetin induced G2/M cell cycle arrest in osteosarcoma cells

**Fig. 2:**
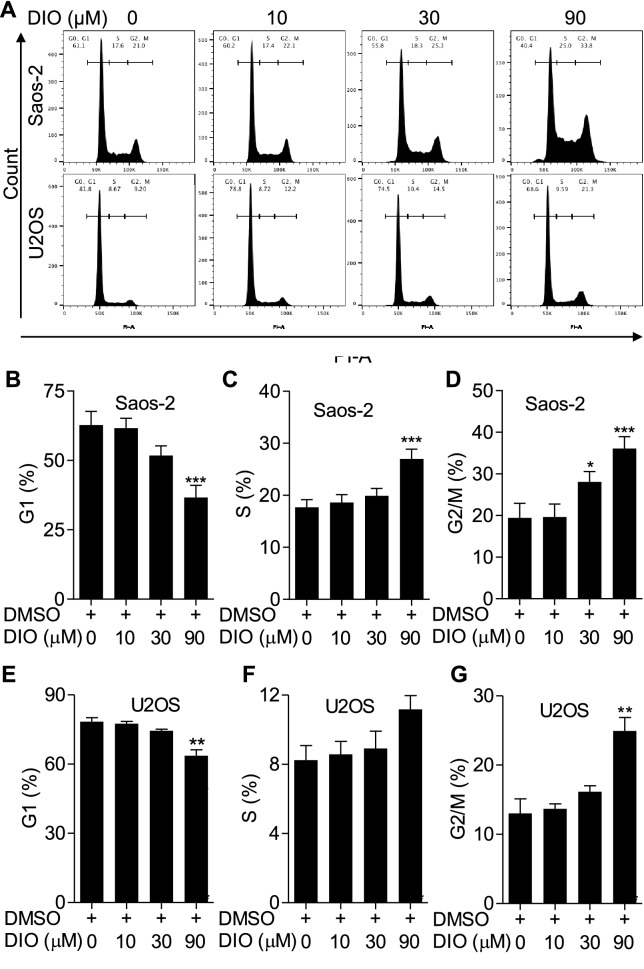
Diosmetin induced G2/M cycle arrest in Saos-2 and U2SO cells. **A** Representative images of the cell cycle distribution in Saos-2 and U2SO cells treated with 0, 10, 30 and 90 μM of diosmetin respectively for 24 h determined by flow cytometry. **B**–**G** Quantification of the percentage of phase population (G1, S, G2/M) in diosmetin treated Saos-2 and U2SO cells. The results were shown as the mean ± SD of three independent experiments (n = 3, *P < 0.05, **P < 0.01 and ***P < 0.001 vs. DMSO control)

Cell cycle progression is one of the crucial factors in cell proliferation [19]. In order to identify whether diosmetin induced cell-cycle inhibition in osteosarcoma cells, we detected the cell cycle distribution of Saos-2 and U2SO cells using PI staining followed by flow cytometry. As showed in Fig. 2A, B, and E, the percentages of both Saos-2 and U2SO cells in the G1 phase decreased significantly when treat by 90 μM diosmetin for 24 h, compared to those of DMSO-treated control cells in the G1 phase. Whereas remarkable increases in the proportion of G2/M phase were observed in Saos-2 cells exposed to 30 or 90 μM diosmetin and U2SO cells exposed to 90 μM diosmetin compared to that of DMSO-treated control groups (Fig. 2A, D, and G). These results demonstrated that diosmetin arrested cell cycle at G2/M phase and blocked the cycle progression of the two lines of osteosarcoma cells, therefore exerted anti-proliferation activity in osteosarcoma cells.

### Diosmetin promoted the apoptosis of osteosarcoma cells

**Fig. 3:**
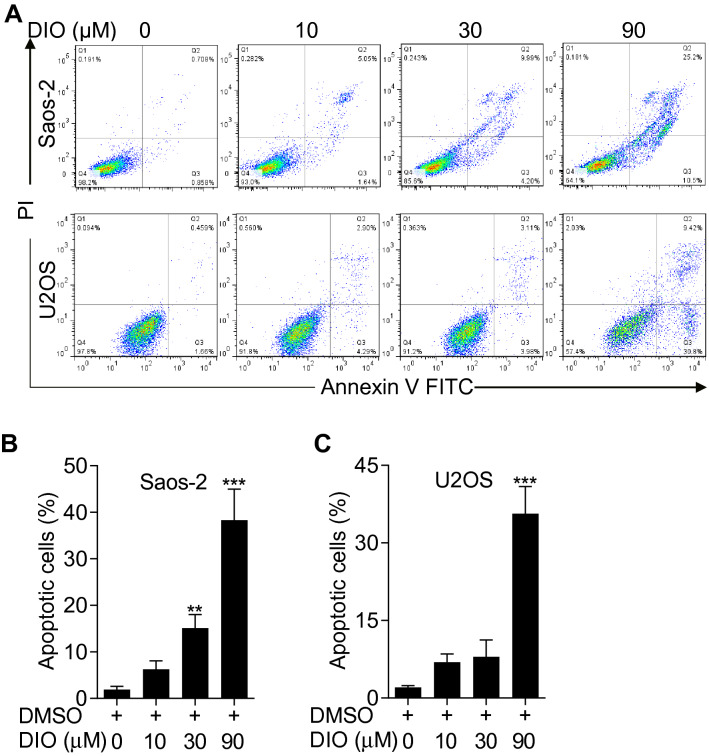
Diosmetin induced apoptosis in osteosarcoma cells. **A** Flow cytometric analysis of apoptotic osteosarcoma cells. Saos-2 or U2SO cells were stained with Annexin V-FITC/PI after diosmetin (0, 10, 30 and 90 μM) treatment for 24 h and cell apoptosis were measured using flow cytometry. **B** and **C** Quantification of the percentage of apoptotic Saos-2 (**B**) and U2SO (**C**) cells after treated with diosmetin at various concentrations for 24 h. The results are shown as the mean ± SD of three independent experiments (n = 3, *P < 0.05, **P < 0.01 and ***P < 0.001 vs. DMSO control)

Deregulation of the cell cycle has been shown to induce mitotic catastrophe and might be involved in triggering apoptosis [20]. Therefore, we performed the Annexin V-FITC/PI double staining with flow cytometry analysis on diosmetin-treated Saos-2 and U2SO cells to determine whether diosmetin induced apoptosis in osteosarcoma cells. Saos-2 or U2SO cells were incubated with0, 10, 30 and 90 μM diosmetin respectively for 24 h, and were then subjected to Annexin V-FITC/PI staining, followed by flow cytometer analysis. When treated with diosmetin, both Saos-2 and U2SO cells demonstrated gradually increased proportion of apoptotic cells, whether in early stage (lower-right quadrant) or in the late stage (upper-right quadrant), along with increasing diosmetin concentration (Fig. 3A). Statistical analysis revealed diosmetin treatment at 90 μM remarkably aggravated apoptosis, with the proportion of apoptotic cells up to 40.2 ± 1.0% and 35.7 ± 1.5% in Saos-2 and U2SO cells (Fig. 3B and C), respectively. These data indicated that diosmetin promoted the apoptosis of osteosarcoma cells.

### Diosmetin upregulated pro-apoptotic proteins while downregulate anti-apoptotic proteins

**Fig. 4:**
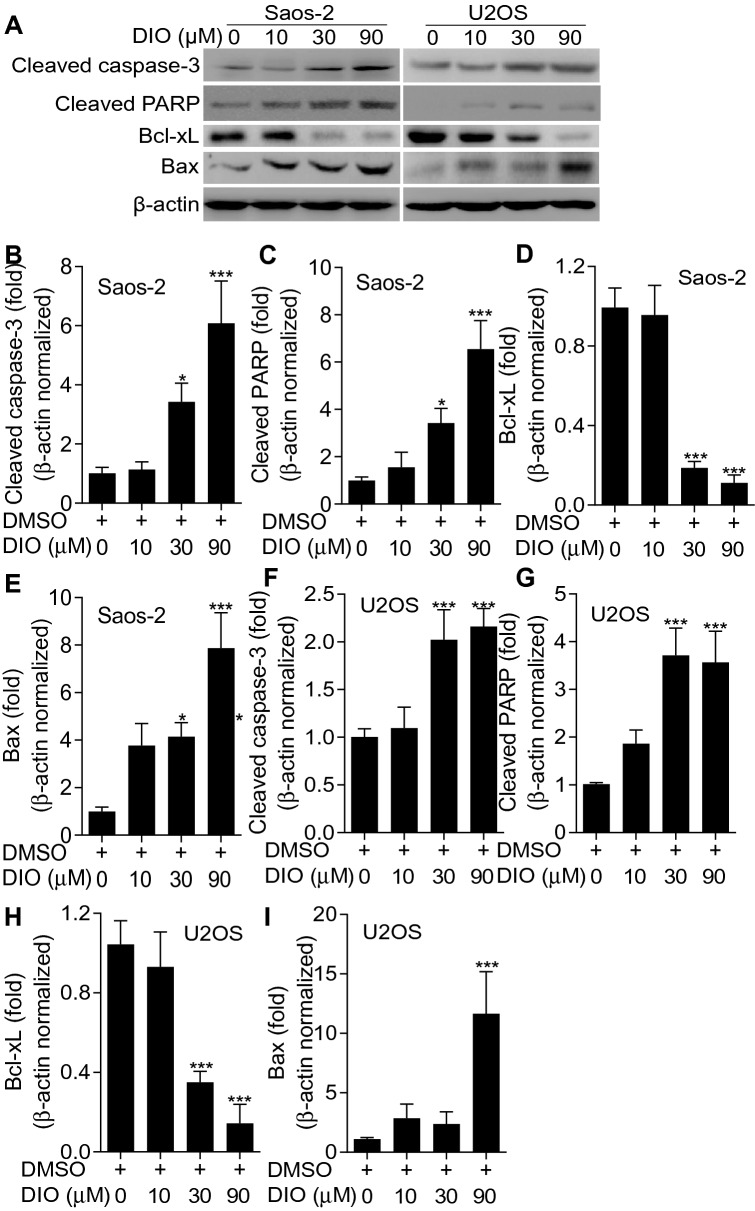
Diosmetin promoted the expressions of pro-apoptotic proteins while reduced the expressions of anti-apoptotic proteins. **A** The expression of apoptotic proteins cleaved Caspase-3, cleaved PARP, Bcl-xL and Bax in Saos-2 and U2SO cells detected by western blot after diosmetin (0, 10, 30 and 90 μM) treatment for 24 h. **B**–**I** Quantification of related proteins by Image J software. The results are shown as the mean ± SD of three independent experiments (n = 3, *P < 0.05, **P < 0.01 and ***P < 0.001 vs. DMSO control)

In order to further confirm the pro-apoptotic effects of diosmetin on osteosarcoma cells, apoptosis associated proteins were detected with western blot. As shown in Fig. 4, the levels of pro-apoptotic proteins including cleaved Caspase-3 (Fig. 4A, B, and F), cleaved-PARP (Fig. 4A, C, and G) and Bax (Fig. 4A, E, and I) significantly increased while the level of the anti-apoptotic protein Bcl-xL (Fig. 4A, D, and H) significantly decreased in both Saos-2 and U2SO cells following the treatment of diosmetin with incremental doses. These results revealed that diosmetin upregulate pro-apoptotic proteins while downregulate anti-apoptotic proteins.

### Diosmetin inhibited STAT3 activation and the expression of its downstream protein c-Myc

**Fig. 5:**
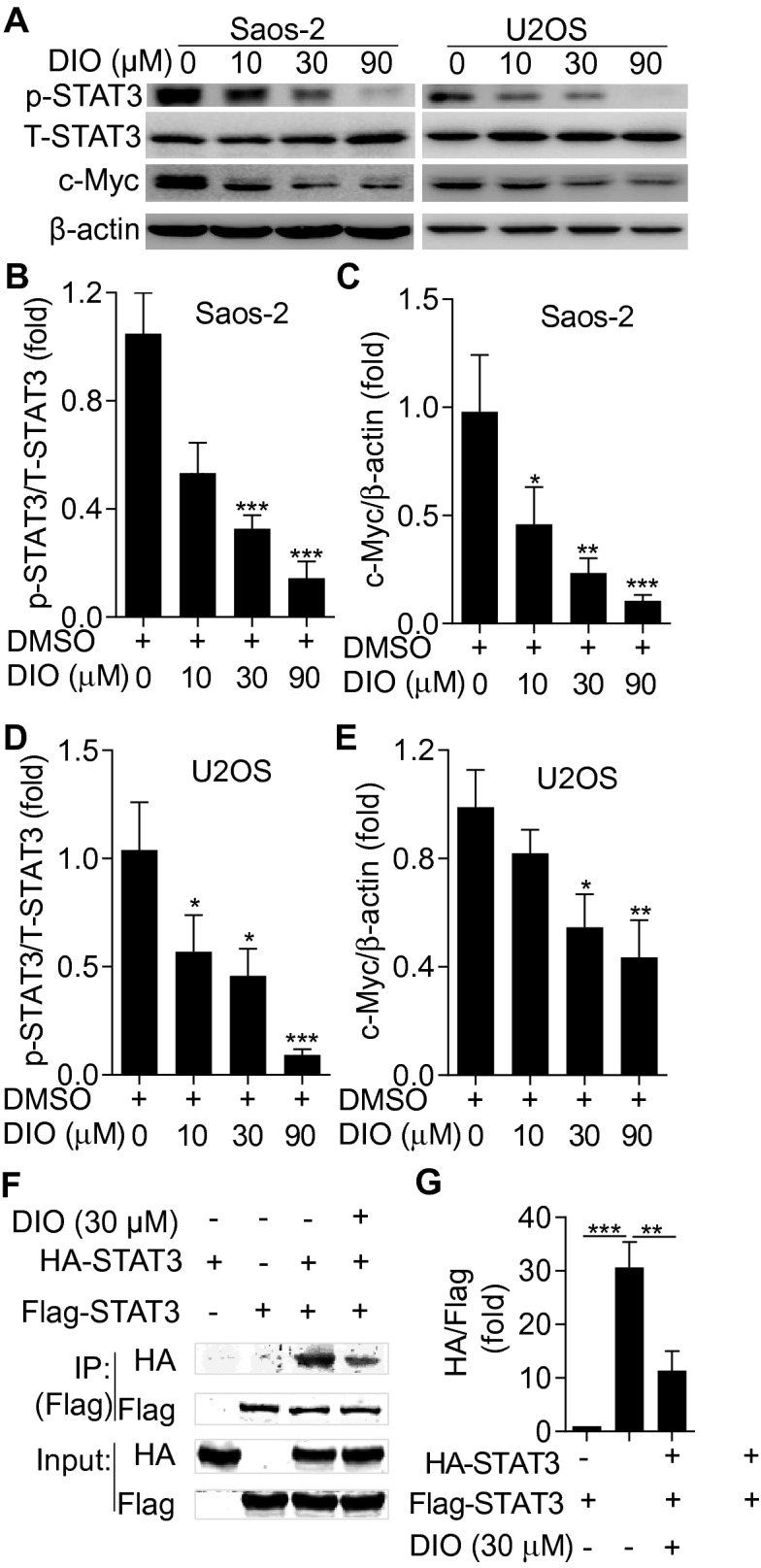
Diosmetin suppressed STAT3 activation and c-Myc expressions in Saos-2. **A** The expression of STAT3 and phosphorylated STAT3 (p-STAT3) and its downstream protein c-Myc in Saos-2 and U2SO cells detected by western blot at 24 h after diosmetin (0, 10, 30 and 90 μM) treatment. **B**–**E** Quantification of related proteins by Image J software. **F** Diosmetin suppressed the activation of STAT3 in osteosarcoma cells. Saos-2 cells transfected with HA-STAT3 and Flag-STAT3 expression plasmid were either left uninduced or stimulated with 30 μM diosmetin for 24 h, and STAT3 was immunoprecipitated from cell lysates with a Flag-STAT3 antibody, separated by SDS-PAGE, analyzed by western blot with a HA-STAT3 antibody. **G** Quantification of the proteins from the immunoprecipitation by Image J software. The results are shown as the mean ± SD of three independent experiments (n = 3, *P < 0.05, **P < 0.01 and ***P < 0.001 vs. DMSO control)

STAT3 is broadly hyperactivated in hematologic malignancies, head and neck cancers, leukemia and a subset of solid tumors and oncogenesis and promotes oncogenesis by up-regulating the expression of target proteins associated with cell proliferation, differentiation, apoptosis (Bcl-2, Bcl-xL), angiogenesis, metastasis and immunity [3–7]. To determine whether diosmetin induced cell apoptosis by suppressing STAT3 signal pathway, we investigated the effects of diosmetin on the expression of STAT3 and its downstream oncoprotein c-Myc in Saos-2 and U2SO cells. The results showed a dose-dependent inhibition of STAT3 phosphorylation in diosmetin-treated Saos-2 and U2SO cells (Fig. 5A, B, and D). Simultaneously, the expression level of c-Myc in diosmetin-treated group also reduced in a dose-dependent manner (Fig. 5A, C, and E). These results suggested that it might account for inhibiting the JAK-STAT3 signaling pathway that diosmetin induced apoptosis in Saos-2 and U2SO cells.

To further verify the dependent role of STAT3 signaling in the cellular response elicited by diosmetin, osteosarcoma cells transfected with HA-STAT3 and Flag-STAT3 expression plasmid were either left uninduced or stimulated with 30 μM diosmetin for 24 h, and STAT3 was immunoprecipitated from cell lysates with a Flag-STAT3 antibody, separated by SDS–PAGE, analyzed by western blot with a HA-STAT3 antibody. As anticipated, the interaction between Flag-STAT3 and HA-STAT3, which reflected STAT3 dimerization and the activation of STAT3 signaling [6], was significantly impeded by diosmetin (Fig. 5F and G). These observations indicated that diosmetin suppressed the activation of STAT3 signaling pathway that resulted in the osteosarcoma inhibition.

### IL-6 treatment rescued and WP1066 imitated the anti-proliferation effect of diosmetin

**Fig. 6:**
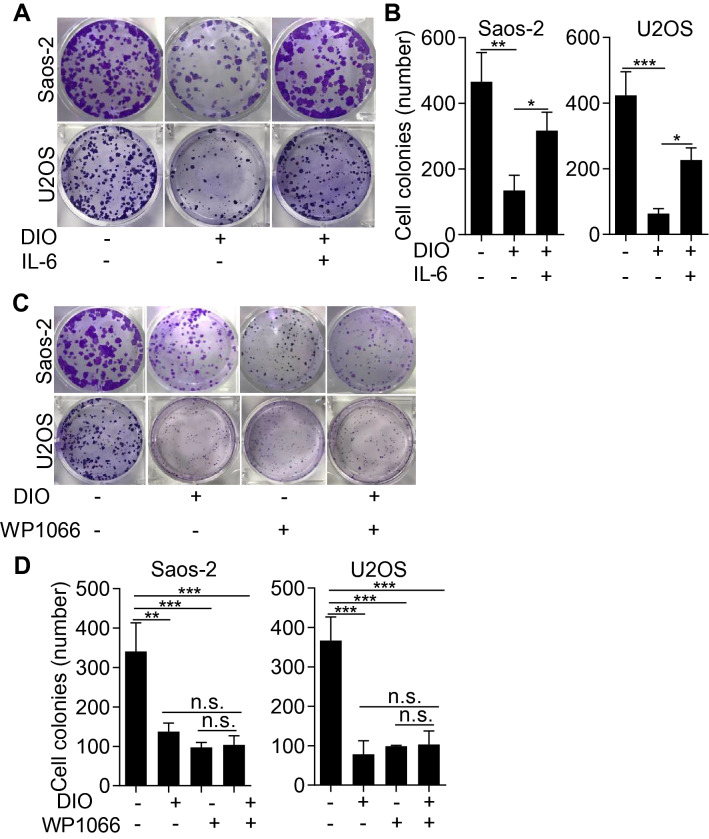
IL-6 treatment counteracted and wp1066 imitated the anti-proliferative activity of diosmetin in osteosarcoma Saos-2 and U2SO cells. Saos-2 or U2SO cells were treated with diosmetin with or without IL-6 for 24 h, replaced with fresh culture medium and cultured for 2 weeks, stained with crystal violet and photographed. **A** Representative images of colonies of Saos-2 and U2SO cells from six-well plates in which cells were treated with DMSO control, diosmetin or diosmetin + IL-6 using colony formation assay. **B** Quantification of colony number in DMSO-, diosmetin- or diosmetin + IL-6-treated Saos-2 and U2SO cells. **C** Representative images of colonies of Saos-2 and U2SO cells from six-well plates in which cells were treated with DMSO control, WP1066, diosmetin or WP1066 + diosmetin using colony formation assay. **D** Quantification of colony number in DMSO-, WP1066, diosmetin- or WP1066 + diosmetin—treated Saos-2 and U2SO cells. The results are shown as the mean ± SD of three independent experiments (n = 3, *P < 0.05, ***P < 0.001)

STAT3 hyperactivation in tumor cells were generally induced by elevated IL-6 in the tumor microenvironment in the majority of human cancers [6]. To determine the involvement of STAT3 signaling in the anti-tumor effects of diosmetin on osteosarcoma cells, we examined the proliferation ability of Saos-2 and U2OS cells by applying diosmetin (0.3 μM) with or without IL-6 (20 ng/mL). As shown in Fig. 6A and B, diosmetin markedly inhibited the proliferation ability of osteosarcoma cells, while IL-6 administration, which could simulate STAT3 signaling activation, counteracted diosmetin-induced anti-osteosarcoma effect and significantly, although partly, reverse proliferation of Saos-2 or U2OS cells. Furthermore, WP1066, a well-known inhibitor for STAT3 activation, effectively inhibited the proliferation of Saos-2 or U2OS cells and 0.1 μM WP1066, when used alone, showed similar antiproliferative effect as that of diosmetin at 0.3 μM (Fig. 6C and D). However, the combination of diosmetin and WP1066 did not significantly increase the antiproliferative effects of each other (Fig. 6C and D). These results, together with the finding that diosmetin significantly impeded STAT3 dimerization and the activation of STAT3 signaling revealed that the anti-osteosarcoma effects of diosmetin were mediated by impeding the activation of STAT3 signaling pathway.

## Discussion

**Fig. 7:**
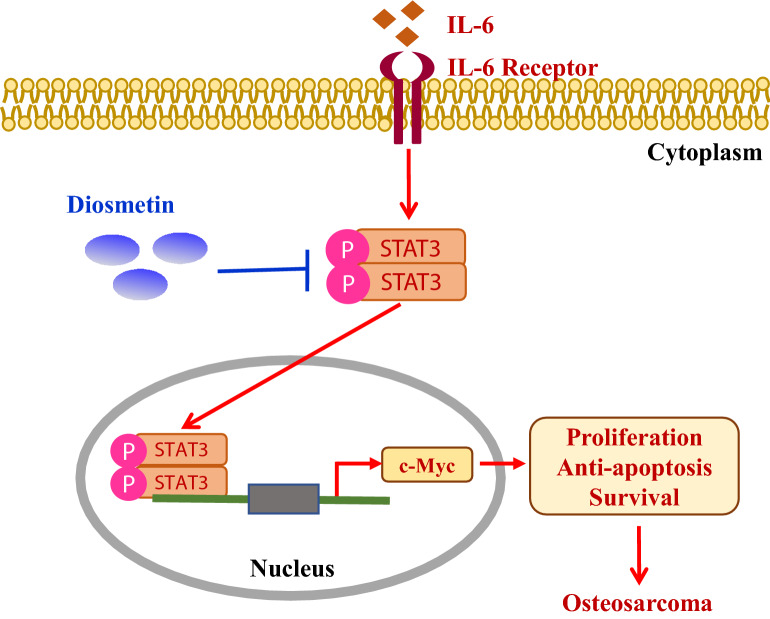
Schematic illustration of the anti-tumor activity of Diosmetin on human osteosarcoma cells by inhibiting the activity of STAT3/c-MYC signaling. Persistent STAT3 activation resulting from the activation of upstream growth factor receptors (including IL-6 receptor), leads to abnormal proliferation and tumorigenesis in human osteosarcoma cells. Diosmetin inhibits cell proliferation and promotes apoptosis through STAT3/c-Myc signaling pathway in human osteosarcoma cells

In this study, we demonstrated that diosmetin, a natural flavonol-type flavonoid, significantly suppressed the cell proliferation of human osteosarcoma cells in both Saos-2 and U2SO lines as well as caused an obvious cell cycle arrest which mainly occurred in the G2/M phase. Meanwhile, we disclosed diosmetin could remarkably promoted cell apoptosis with reduced anti-apoptotic protein Bcl-xL while elevated pro-apoptotic proteins cleaved Caspase-3, cleaved-PARP and BAX. Furthermore, we determined that the anti-osteosarcoma activity of diosmetin was possibly attributed to suppressed activation of STAT3/c-Myc signaling pathway as it dose-dependently inhibited STAT3 phosphorylation and reduced c-Myc expression as well as inhibited STAT3 dimerization while its anti-proliferation effect was impeded by IL-6 by driving the activation of STAT3/c-Myc pathway (Fig. 7). These observations illustrated the effect of diosmetin on inhibiting cell proliferation, inducing G2/M cell cycle arrest and apoptosis via STAT3/c-Myc signaling pathway, which suggesting that it might be a new potential candidate for osteosarcoma treatment. Osteosarcoma is a primary bone tumor in young patients with high malignancy and poor prognosis. Survival for patients with osteosarcoma have not changed in several decades despite extensive translational and clinical investigation. Therefore, innovative modes of therapy which exploit novel targets are desperately needed to improve outcome, especially for metastatic or recurrent patients [1]. As one of major flavones found naturally in citrus fruit, diosmetin has a variety of pharmacological effects, such as anti-inflammatory, antioxidant and antinociceptive activities. But it almost has no toxicity and side effect [16]. Our work revealed the anti-osteosarcoma effect of diosmetin on human Saos-2 and U2SO osteosarcoma cells for the first time, providing a promising chemotherapy agent in treating osteosarcoma.

STAT3 is a transcriptional factor that is primarily activated by IL-6 family cytokine receptor-associated Janus kinases (JAKs) and had a prominent role in regulating the proliferation, survival, invasion, and metastasis of tumor cells as well as immunosuppression [3]. When activated, phosphorylated STAT3 dimerized and translocated into the nucleus where it bonded to consensus response elements in the promoters of target genes, regulating the transcription of several genes which was associated with cellular proliferation (such as c-Myc, Bcl-2 and cyclins) and survival (such as BCL-xL and survivin) [21]. Previous studies have confirmed that STAT3 is involved in a variety of cellular pathways, such as proliferation, stress resistance, differentiation and apoptosis. Uncontrolled cell proliferation and resistance to cell death often leads to tumorigenesis while suppressing cell proliferation and inducing apoptosis has been regarded as popular strategies in controlling cancer progression [22]. In our study, diosmetin inhibited tumor cell proliferation and promoted apoptosis as well as suppressed STAT3/c-Myc signaling pathway in osteosarcoma cells, which is consistent with the inhibitory effects of another STAT3 inhibitor toosendanin on cell proliferation of osteosarcoma 143B Cells [23]. Using flow cytometry, we also showed that blocked the cycle progression and arrested cell cycle at G2/M phase in both Saos-2 and U2SO osteosarcoma cells. Abnormal cell cycle regulation is an important reason for tumorigenesis. Dysregulated cell cycle would cause uncontrolled cell proliferation while permitting cells obtain the ability to replicate and grow without limitation [24]. G2/M phase regulation point is the key point in cell cycle regulation for signal transmission inside and outside the cell, integration and collection to the nucleus to regulate cell proliferation. Thus, cell cycle arrest might serve as one way that diosmetin inhibiting the cell proliferation of osteosarcoma. Moreover, our study also revealed that diosmetin downregulated anti-apoptotic protein Bcl-xL while upregulated pro-apoptotic proteins such as cleaved Caspase-3, cleaved-PARP and Bax in osteosarcoma Saos-2 and U2SO cells, together with the suppression of p-STAT3 and c-Myc, suggesting that STAT3/c-Myc signaling pathway might be involved in the anti-proliferative and pro-apoptotic effects of diosmetin. Besides STAT3/c-Myc signaling pathway, various signaling pathways, including RANKL (receptor activator of nuclear factor-κB ligand)/ RANK (receptor activator of nuclear factor-κB) and Wnt signalings, were also involved in the tumorigenesis of osteosarcoma [25, 26]. In previous studies, Shao et al*.* [27] found that diosmetin could inhibit osteoclastic formation induced by RANKL, which is a classical signal pathway that regulating the differentiation of osteoclasts. Wnt signaling, which controls multiple cellular processes, including proliferation, cell fate determination, and differentiation, is a critical pathway in osteosarcoma progression [25]. Therefore, diosmetin, to some extent, exerts anti-osteosarcoma effects partly via signaling pathways (such as Wnt and RANKL/RANK), in addition to dramatically inhibiting the activation of STAT3/c-Myc signaling pathway. It has been reported that silencing of ERβ promotes the invasion and migration of osteosarcoma cells via activating Wnt signaling pathway [28] and the activation of STAT3 signaling up-regulates ERβ expression in lung cancer cells [29]. Diosmetin has been reported to act as an agonist for estrogen receptor-β (ERβ), and it facilitates osteoblast differentiation and suppresses the production of sclerostin, the anti-osteoblastogenic Wnt inhibitor, in osteoblasts [30]. Moreover, diosmetin has been found to act as a weak agonist for TrkB receptor [31], which plays a vital role in osteosarcoma pathology [32]. In the present study, diosmetin shows anti-osteosarcoma effects by inhibiting the activation of STAT3/c-Myc signaling pathway, instead of relying on ERβ or TrkB signaling pathways. How diosmetin affects ERβ or TrkB signaling pathways and their crosstalk with STAT3/c-Myc signaling pathway to inhibit osteosarcoma need to be further explored.

Hyperactivation of STAT3 is primarily associated with the promotion of tumor growth although it was reported that activated STAT3 might have tumor-suppressive functions in Kras-induced lung cancer in *Apc*-mutant mice [3]. Specifically, a growing body of evidence indicated that STAT3 played a key role in the development and progression of osteosarcoma and it was commonly activated in human osteosarcoma cell lines as well as clinical osteosarcoma tissues [33, 34]. In addition, STAT3 activation was reported to promote mesenchymal stem cells-induced osteosarcoma cell survival and drug resistance while blocking STAT3 signaling revert resistance to chemotherapy in osteosarcoma Saos-2 cells to drug treatment [35]. Therefore, STAT3 is deemed as an attractive target for chemotherapy of tumors, including osteosarcoma [8, 36]. Several STAT3 inhibitors such as AZD9150, C188-9, OPB-31121, OPB-51602 and C188-9 have now reached clinical trials [3]. So far there is nearly no anti-tumor drug directly acting on the STAT3 on the market except napabucasin, which was recently approved for pancreatic cancer and esophageal tumor treatment [37]. Naturally, fairly poor agents on STAT3 have been developed for osteosarcoma treatment. In this study, we demonstrated that the administration of diosmetin on osteosarcoma cells like Saos-2 and U2SO cells significantly inhibited cell proliferation while promoted apoptosis, with remarkable reduction of STAT3, p-STAT3 and its downstream protein c-Myc levels in a dose-dependent manner. Similar results were found when STAT3 signal pathway was blocked with STAT3 inhibitors (FLLL32, S31-201 and toosendanin) or shRNA targeting STAT3 [22, 33, 38]. Accumulating studies demonstrate that IL-6 plays a critical role in the growth and progression of tumors by binding to IL-6 receptor and activating STAT3 signaling pathway. In the present study, we showed that IL-6 treatment rescued the anti-proliferation effect of diosmetin and immunoprecipitation also showed STAT3 dimerization (activation) was significantly suppressed by diosmetin, indicating the involvement of STAT3 activation (phosphorylation and dimerization) in cell proliferation and apoptosis of osteosarcoma cells, and blockade of the STAT3 pathway may be a therapeutic strategy against osteosarcoma. Given IL-6 is the main upstream activator, but not the downstream target of STAT3, it is not sufficient to to validate the effects of diosmetin on the STAT3 / c-MYC signaling pathway and cell proliferation of osteosarcoma cells by the reversal effects of IL-6 that drives the activation of STAT3/c-Myc pathway. So, the role of WP1066, a well-known inhibitor for STAT3 activation, in cell proliferation of osteosarcoma cells was also compared with that of diosmetin. As diosmetin, WP1066 (0.1 μM) effectively inhibited the proliferation of U2OS cells at similar level that diosmetin (0.3 μM) did. However, the combination of diosmetin and WP1066 did not significantly increase the antiproliferative effects of each other. These data, together with the founding that diosmetin significantly impeded STAT3 dimerization and the activation of STAT3 signaling, confirm that diosmetin, like WP1066, inhibited osteosarcoma by impeding the activation of STAT3 signaling pathway. It should be noted that STAT3 is phosphorylated by the action of Janus kinases (JAKs) in response to cytokines or growth hormones in addition to IL-6, and WP1066 also inhibits Jak2/STAT3 activation induced by cytokines and STAT5 in addition to the inhibitory effect on STAT3 activation [39]. These need be addressed in the future research.

Diosmetin is a type of common flavonoids. It is rich in citrus fruits, olive leaves and extracts from common herbs in southern China with a variety of functions such as scavenging free radicals, anti-inflammatory and anti-oxidative stress [13–15]. Recently, it is shown that diosmetin has anti-tumor effect by inhibiting the cell proliferation, inducing cell apoptosis and regulating cell cycle on breast cancer cells, liver cancer cells, colon cancer cells, leukemia cells and other tumors, indicating it is a potential promising treatment for cancers [17, 18, 40, 41]. Studies have shown that diosmetin exerts anti-tumor effect through a variety of mechanisms and the mechanisms are varied in different tumors. For example, diosmetin inhibited the growth of MDA-MB468 breast cancer cells by the activation of CYP1A1 and CYP1B1, which led to the cell cycle arrested in G1 phase, but it was non-cytotoxicity to normal breast cells MCF-10A, suggesting that the use of diosmetin in cancer treatment may be safe and selectively cytotoxic to cancer cells [17] While in human liver cancer HepG-2 cells, diosmetin induced apoptosis by up-regulating Bax, cleaved-caspase-3, cleaved-caspase-8 and cleaved-caspase-9, and induced G2/M cell cycle arrest by up-regulating the expression levels of p21 and p53, and down-regulated cyclin B, CDK-1 [42]. In addition, diosmetin inhibited the activity of cytochrome P450 enzymes, regulated the TGF-β signal pathway by up-regulating the expression of p53 and down-regulating the protein expression of Bcl-2, TGF-β, TβR-II, Smad-3, p-Smad2/3, and induce HepG2 apoptosis in liver cancer [43]. In this study, we firstly verified the anti-osteosarcoma effect of diosmetin in both Saos-2 and U2SO cell lines and determined it inhibited cell proliferation and promoted apoptosis of human osteosarcoma cells via STAT3/c-Myc signaling pathway. Notably, diosmetin is safe and barely has adverse reaction, suggesting it might be a potential promising therapy for human osteosarcoma with exploiture and deserves further investigation.

In summary, the current study explored the antitumor activity of diosmetin on human osteosarcoma cells and illustrated for the first time that diosmetin inhibited cell proliferation, induced G2/M cell cycle arrest and apoptosis by suppressing STAT3/c-Myc signaling pathway in Saos-2 and U2SO osteosarcoma cells. Therefore, our work provided a promising strategy that diosmetin may be used in clinic practice for the treatment of osteosarcoma and revealed that STAT3 might be a promising therapeutic target for osteosarcoma.

## Data Availability

All the data generated or analyzed during this study is available.

## Abbreviations

DMEM: Dulbecco's modified eagle medium
DMSO: Dimethyl sulfoxide
ECL: Enhanced chemiluminescence
HA: Hemagglutinin
HRP: Horseradish peroxidase
IL-6: Interleukin-6
MTT: 3-(4,5-Dimethylthiazol-2-yl)-2,5-diphenyltetrazolium bromide
PARP: Poly ADP-ribose polymerase
PBS: Phosphate buffered saline
SDS–PAGE: Sodium dodecyl sulfate polyacrylamide gel electrophoresis
STAT3: Signal transducer and activator of transcription 3
TBST: Tris buffered saline with tween 20
